# Estimating Time-Varying PCB Exposures Using Person-Specific Predictions to Supplement Measured Values: A Comparison of Observed and Predicted Values in Two Cohorts of Norwegian Women

**DOI:** 10.1289/ehp.1409191

**Published:** 2015-07-17

**Authors:** Therese Haugdahl Nøst, Knut Breivik, Frank Wania, Charlotta Rylander, Jon Øyvind Odland, Torkjel Manning Sandanger

**Affiliations:** 1 Department of Community Medicine, UiT–The Arctic University of Norway, Tromsø, Norway; 2 NILU–Norwegian Institute for Air Research, Fram Centre, Tromsø, Norway; 3 University Hospital of North Norway, Tromsø, Norway; 4 NILU–Norwegian Institute for Air Research, Kjeller, Norway; 5 Department of Chemistry, University of Oslo, Oslo, Norway; 6 Department of Physical and Environmental Sciences, University of Toronto Scarborough, Toronto, Ontario, Canada

## Abstract

**Background:**

Studies on the health effects of polychlorinated biphenyls (PCBs) call for an understanding of past and present human exposure. Time-resolved mechanistic models may supplement information on concentrations in individuals obtained from measurements and/or statistical approaches if they can be shown to reproduce empirical data.

**Objectives:**

Here, we evaluated the capability of one such mechanistic model to reproduce measured PCB concentrations in individual Norwegian women. We also assessed individual life-course concentrations.

**Methods:**

Concentrations of four PCB congeners in pregnant (*n* = 310, sampled in 2007–2009) and postmenopausal (*n* = 244, 2005) women were compared with person-specific predictions obtained using CoZMoMAN, an emission-based environmental fate and human food-chain bioaccumulation model. Person-specific predictions were also made using statistical regression models including dietary and lifestyle variables and concentrations.

**Results:**

CoZMoMAN accurately reproduced medians and ranges of measured concentrations in the two study groups. Furthermore, rank correlations between measurements and predictions from both CoZMoMAN and regression analyses were strong (Spearman’s *r* > 0.67). Precision in quartile assignments from predictions was strong overall as evaluated by weighted Cohen’s kappa (> 0.6). Simulations indicated large inter-individual differences in concentrations experienced in the past.

**Conclusions:**

The mechanistic model reproduced all measurements of PCB concentrations within a factor of 10, and subject ranking and quartile assignments were overall largely consistent, although they were weak within each study group. Contamination histories for individuals predicted by CoZMoMAN revealed variation between study subjects, particularly in the timing of peak concentrations. Mechanistic models can provide individual PCB exposure metrics that could serve as valuable supplements to measurements.

**Citation:**

Nøst TH, Breivik K, Wania F, Rylander C, Odland JØ, Sandanger TM. 2016. Estimating time-varying PCB exposures using person-specific predictions to supplement measured values: a comparison of observed and predicted values in two cohorts of Norwegian women. Environ Health Perspect 124:299–305; http://dx.doi.org/10.1289/ehp.1409191

## Introduction

Polychlorinated biphenyls (PCBs) have been detected globally and may be harmful to both humans and the environment. Emissions of PCBs began to increase in the 1930s but started to decrease from the 1980s onward following restrictions or bans on their production and use in many countries (Breivik et al. 2010). Exposure of the general population to PCBs has been linked to dietary intake (Caspersen et al. 2013; Darnerud et al. 2006; Rylander et al. 2012), and time trends in human blood have been shown to reflect those of the emissions (Nøst et al. 2013; Quinn and Wania 2012). Although temporal changes in the human body burden of PCBs have been investigated in general terms (e.g., Quinn and Wania 2012), knowledge of such longitudinal body burden age-trends (LBATs) within individuals remains limited. Of particular interest are historical exposures in individuals during sensitive life stages (Verner et al. 2008, 2011).

Human bioaccumulation and PCB body burden as functions of time have been estimated in several pharmacokinetic modeling approaches of varying design and complexity (Alcock et al. 2000; Moser and McLachlan 2002; Ritter et al. 2011; Verner et al. 2008, 2013). In these studies, time-variant human PCB intake is often calculated by extrapolating point estimates of dietary intake back in time based on historical changes in emissions. The CoZMoMAN model (Breivik et al. 2010) also predicts time-variant human concentrations, but these concentrations are linked to historic emissions not by mere scaling but by a mechanistic simulation of environmental fate and human food-chain bioaccumulation. These calculations describe how emissions of contaminants are transported and distributed in the environment, and they predict concentrations in environmental compartments (air, water, soil, sediment) and in the organisms in one aquatic and one agricultural food chain (e.g., grass, cows, fish). Human dietary intake rates are subsequently determined from the time-variant concentrations in air, water, and the tissues of food organisms. Using PCB-153 as an example, CoZMoMAN has previously been used to evaluate *a*) generational differences in prenatal, postnatal, and lifetime exposures (Quinn et al. 2011); *b*) associations with age in different sampling years and study designs (Quinn and Wania 2012); and *c*) the effects of transient dietary changes made by pregnant women on the pre- and postnatal exposure in their children (Binnington et al. 2014). A similar modeling strategy has also been explored to evaluate PCB exposure time trends in Arctic populations with concurrent transitions in dietary habits (Quinn et al. 2012). Such studies can aid in the formulation and testing of hypotheses concerning the impact of emission reductions and changes in lifestyle (e.g., diet) on human PCB exposure.

PCB concentrations predicted by CoZMoMAN were within the range of those measured in environmental compartments, organisms, and humans in Scandinavia (Breivik et al. 2010; Nøst et al. 2013). Furthermore, CoZMoMAN has reproduced PCB contamination time trends from 1979 to 2007 in Norwegian men (Nøst et al. 2013). Previous studies evaluating CoZMoMAN have compared concentrations predicted for a hypothetical “average” person with the observed population means. Individual predictions of PCB concentrations in infants made by a different toxicokinetic model were accurate relative to and strongly correlated with measurements, indicating that such models can produce reliable person-specific predictions (Verner et al. 2013). To our knowledge, such an evaluation has not been performed for adults. Here, we used PCB concentrations measured in pregnant and postmenopausal Norwegian women to evaluate the person-specific predictions of concentrations of four PCBs from mechanistically derived intake rates. In addition, individual LBATs were derived to reconstruct their past exposures. An additional approach using statistical regression analyses of measured PCB concentrations was performed to evaluate the input parameters to CoZMoMAN and to identify any dietary or lifestyle predictors not considered within the model.

## Material and Methods

*Study population.* The subjects included in the present study were *a*) pregnant women (*n* = 515) in the Northern Norway mother-and-child contaminant cohort study (MISA), who were enrolled during the second trimester of pregnancy and donated a blood sample during 2007–2009, and *b*) postmenopausal women (*n* = 311) from the general Norwegian population who were participants in the Norwegian women and cancer study (NOWAC) and donated blood samples in 2005. The median (range) ages at time of blood sampling for the MISA women and the NOWAC women were 30 (18–43) and 56 (48–62) years, respectively. Details and population characteristics for the MISA and NOWAC studies have been described by Veyhe et al. (2012) and Waaseth et al. (2008), respectively. The studies were approved by the Regional Committees for Medical Research Ethics, and all participants provided written informed consent.

Demographic, dietary, and lifestyle variables were extracted from questionnaires, and information on births for the MISA women was extracted from the Norwegian Birth Registry. Daily intake (grams per day) of a range of food items had been calculated from food-frequency questionnaires for the MISA women (Veyhe et al. 2012) and the NOWAC women (Skeie et al. 2006). The food-frequency questionnaires in the NOWAC study have been validated using several approaches (Hjartåker et al. 1997, 2007; Parr et al. 2006), and the questionnaire in the MISA study was expanded from the NOWAC questionnaires. Some questions were unique to the study groups, for example, intake of seagull eggs was only included in the MISA study.

*Chemical analyses.* Concentrations of PCBs in the MISA women have been reported by Veyhe et al. (2015), and those in the NOWAC women have been reported by Rylander et al. (2012). The methods employed for the PCB analyses in the MISA and NOWAC studies were similar and have been described in detail by Hansen et al. (2010) and Rylander et al. (2012), respectively. Briefly, internal standards, formic acid, and deionized water were added to either 2 mL serum (MISA samples) or 0.75 g plasma (NOWAC samples) and stored in a refrigerator at 4°C overnight before being extracted using a solid phase extraction column with dichloromethane as the eluting solvent. Further clean-up involved elution of compounds from solid-phase extraction columns [Florisil (Fisher, Pittsburgh, PA), 1 g, deactivated] with *n*-hexane/dichloromethane. PCBs in the extracts were identified and quantified with a gas chromatograph/mass spectrometer operated in electron impact mode. Assessment of isotopic mass ratios, blank samples, and standard reference materials ensured the quality of the PCB results.

Lipids were determined enzymatically, and the summed amount of lipids was calculated as follows: Total lipids = 1.677(total – free cholesterol) + free cholesterol + triglycerides + phospholipids (Akins et al. 1989).

*Time-variant model simulations of PCB concentrations.* The CoZMoMAN parameterization of the calculation of time-variant PCB contamination of air, water, soil, and sediments and of organisms comprising the agricultural and aquatic food chains for the time period from 1930 to 2010 from historical emissions was identical to that described in Breivik et al. (2010). CoZMoMAN was used to predict time-variant person-specific PCB concentrations for each woman from birth until time of sampling by running the model one time for each person. The model was supplied with person-specific parameters for the woman’s year and date of birth, the duration of breastfeeding for each child (maximum four), and the woman’s daily intake of meat (grams lipid weight), dairy products (grams lipid weight), and fish (grams fresh weight), of which the last was assumed to be 35% piscivorous fish (“cod”) and 65% planktivorous fish (“herring”) (Nøst et al. 2013). The input information is summarized in Table 1.

**Table 1:** Summary of input information for CoZMoMAN simulations of MISA (*n* = 310) and NOWAC (*n* = 244) women.

Variable	MISA			NOWAC		
	Median	Minimum	Maximum	Median	Minimum	Maximum
Birth year	1977	1965	1991	1949	1943	1957
Number of children	1	0	4^*a*^	2	0	4^*a*^
Age at 1st child	26	—	41	23	—	39
Age at 2nd child	28	—	37	27	—	40
Age at 3rd child	30	—	36	30	—	38
Age at 4th child	32	—	33	32	—	41
Months of breastfeeding of 1st child	12	—	36	4	—	36
Months of breastfeeding of 2nd child	12	—	38	5	—	26
Months of breastfeeding of 3rd child	13	—	22	6	—	36
Months of breastfeeding of 4th child	15	—	30	6	—	30
Daily intake of fish (grams fresh weight/day)	51.2	3.29	137	75.4	0.86	199
Daily intake of dairy products (grams lipid/day)	11.1	2.02	44.8	12.0	0.99	70.7
Daily intake of meat (grams lipid/day)	16.2	2.83	34.9	15.0	1.52	33.7
^***a***^The maximum number of children that could be included in the model estimations was four.						

Individual dietary intake rates were calculated from the questionnaire responses addressing the consumption of different food groups during the preceding year. Individual food items were classified as fish, meat, or dairy and were summed accordingly. Contributions from mixed products were estimated based on percentage contents in common brands. The lipid content of meat and dairy food items was obtained from the Norwegian Food Composition Table (Norwegian Food Safety Authority 2014). Wet weight intakes of fish liver (consumed by 101 MISA women and 63 NOWAC women) were multiplied by a factor of 50 because of the high lipid content of this tissue (Norwegian National Institute of Nutrition and Seafood Research 2014) relative to the model-assumed lipid content of 0.5%, which represents fish muscle (Czub and McLachlan 2004). The summed daily intakes were similar to those reported for the study groups in Veyhe et al. (2012) and Rylander et al. (2012). An age-dependent ingestion rate, I_default_(X), is assumed within CoZMoMAN (Czub and McLachlan 2004 and references therein). Because the input intake at 25 years, I_individual_(25), must be specified as the model input, the estimated individual intake rate at the time of sampling at age X, I_individual_(X), was adjusted to age 25 according to Equation 1:

I_individual_(25) = I_default_(25) × I_individual_(X)/I_default_(X). [1]

The reported total number of months of exclusive and partial breastfeeding for each child was selected to represent the duration of breastfeeding in the simulations. The women’s body weight, metabolic rate, and lipid mass were not described by person-specific information but by the default parameterization (Breivik et al. 2010).

To evaluate the importance of person-specific parameterization of input variables to the predictive ability of CoZMoMAN, we performed additional simulations for PCB-153 in which individual input values were disregarded and replaced with group median or fixed values for all individuals; see Supplemental Material, Table S1.

*Data treatment and statistical methods.* Statistical analyses were performed using R (v3.0.0; R Core Team 2013), and statistical significance was defined as *p* < 0.05. Almost all PCB concentrations had lognormal distributions as evaluated by Shapiro–Wilk tests (data not shown).

The evaluation of CoZMoMAN-predicted and measured concentrations of PCB-153 included 310 and 244 individuals from the MISA and NOWAC studies, respectively. We excluded individuals who had incomplete information sets (questionnaire, PCB measurement, and details about births, *n* = 116) or > 4 children (*n* = 8). Furthermore, women who stated that they did “not eat a Norwegian diet on a regular basis” (*n* = 39) or that they consumed food items known to be heavily contaminated (seagull eggs, MISA women only, *n* = 59) that are not considered by CoZMoMAN were also excluded.

Predicted and measured concentrations were compared using scatter plots and by calculating Spearman’s rank (*r_s_*) and Pearson’s (*r_p_*) correlation coefficients (log-transformed for Pearson’s correlation). In addition, measured and predicted values were divided into quartiles, and the weighted Cohen’s κ was subsequently calculated as a measure of inter-method agreement for the quartile categorization.

To evaluate systematic discrepancies (individual concentration deviations) between measurements and CoZMoMAN predictions, we assessed potential relationships between discrepancies and input parameters as well as dietary and lifestyle variables not accommodated by CoZMoMAN. To further identify potential influential predictors not accommodated by CoZMoMAN, a statistical approach employing linear regression models was used to examine relationships between measured PCB concentrations and all dietary and lifestyle variables (305 and 165 in MISA and NOWAC, respectively) from the questionnaires. Principal component analyses that included dietary and lifestyle variables were initially conducted for each study group (results not shown) to select potential predictors of concentrations that were further assessed in linear regression models. Models were constructed separately for MISA and NOWAC women, and the best models were selected based on the significance of covariates and pairwise log likelihood tests. Estimated concentrations (hereafter referred to as predictions) were derived for each individual from the coefficients in the best-fitted models. Furthermore, the agreement between regression predictions and measurements was evaluated using the same methods as for the CoZMoMAN predictions.

## Results

*Concentrations of PCBs 118, 138, 153, and 180 in Norwegian women.* Summary statistics for the concentrations of PCBs 118, 138, 153, and 180 have been reported in Veyhe et al. (2015); the medians (range) were 4.0 [< limit of detection (LOD)–38], 14 (2.8–118), 25 (5.3–201), and 16 (3.0–159) ng/g lipid, respectively. Concentrations of the same PCBs in plasma from postmenopausal Norwegian women were reported by Rylander et al. (2012), and median (range) concentrations of PCBs 118, 138/163, 153, and 180 were 14 (< LOD–49), 62 (< LOD–164), 82 (< LOD–211), and 65 (< LOD–182) ng/g lipid, respectively.

**Figure 1:**
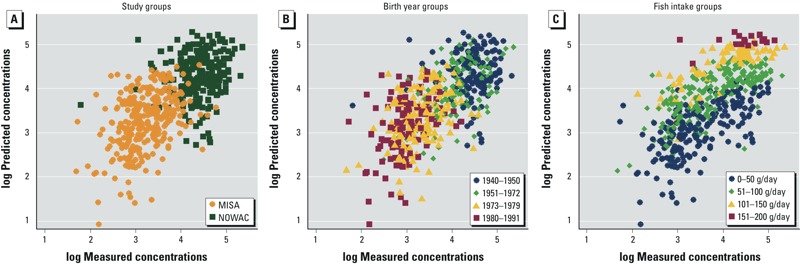
Measured serum concentrations of PCB-153 and those predicted (both in nanograms/gram lipid) by CoZMoMAN for the MISA (*n *= 310) and NOWAC (*n *= 244) women according to their (*A*) study group, (*B*) birth year, and (*C*) daily intake of fish.

*Evaluation of concordance between measurements and predictions.* Comparisons of concentrations predicted by CoZMoMAN with those measured in the MISA and NOWAC women are displayed for PCB-153 in Figure 1A and Table 2; for PCBs 118, 138, and 180, see Supplemental Material, Figure S1 and Table S2.

**Table 2:** Median predicted concentrations of PCB-153 (nanograms/gram lipid) and their discrepancies with and ratios and correlation to measured concentrations.

Study group	Median prediction	Median discrepancy	Median ratio (range)	Correlation *r*_*s*_	Correlation *r*_*p*_^*a*^
MISA	28.8	+4.8	1.06 (0.16–6.44)	0.40**	0.41**
NOWAC	78.2	–3.3	0.94 (0.16–7.92)	0.13**	0.14*
Combined	—	—	—	0.67**	0.67**
^***a***^*r*_*p*_ calculated for log-transformed concentrations. *p < 0.05. **p < 0.001.					

Systematic discrepancies between predicted and measured concentrations are shown in Figure 1B,C; see also Supplemental Material, Table S3. The estimated daily fish intakes, birth year, and duration of breastfeeding were associated with discrepancies between the CoZMoMAN predictions and the measured values. Replacing individual values with median daily intakes of meat, fish, and dairy products increased correlations from those observed when individual information was used for the MISA women; however, this substitution resulted in unrealistically narrow predictions for the NOWAC women (see Supplemental Material, Figure S2). For all other hypothetical simulations that disregarded individualized parameterization, the correlation to the measured concentrations was lower than the main simulation that included fully individualized input parameterization (see Supplemental Material, Table S1). Furthermore, the correlation between measured and predicted concentrations was better for parous women (*r_s_* = 0.67, *p* < 0.0001, *n* = 157 MISA women and 234 NOWAC women) than for nulliparous women (*r_s_* = 0.44, *p* < 0.0001, *n* = 156 MISA women and 10 NOWAC women).

*Predictions of PCB-153 concentrations from linear regression models.* Linear regression models including any significant covariates from questionnaire information were evaluated, and the best-fitted models explained 36% and 22% of the variation in the measured concentrations (see Supplemental Material, Table S4). The year of the participant’s birth and duration of breastfeeding were significant predictors for both study groups. Additionally, body weight and daily intake of fish liver and freshwater fish were significant predictors for MISA women. Predictions derived from these models correlated with measurements with *r_s_* = 0.65, *p* < 0.0001 for the MISA women and *r_s_* = 0.52, *p* < 0.0001 for the NOWAC women (see Supplemental Material, Figure S3). Furthermore, the median predicted concentrations were in accord with the measured values: 27.1 (predicted) and 24.3 (measured) ng/g lipid for MISA women, and 85.4 (predicted) and 80.5 (measured) ng/g lipid for NOWAC women.

*Concordance of quartile categorization.* Table 3 shows the number of individuals assigned to the correct quartile based on both CoZMoMAN predictions and regression analyses in addition to the weighted Cohen’s κ as a measure of agreement in quartile categorization from measurements and predictions. The agreement was stronger for predictions from the regression models than for the mechanistic model. Furthermore, the agreement was stronger when the results for both study groups were combined, and better for the MISA group than for the NOWAC group.

**Table 3:** Agreement in quartile categorization based on a comparison of predictions obtained from CoZMoMAN and linear regressions with the measured concentrations in MISA and NOWAC women.

Approach	Correct quartile *n* (percent)			Weighted Cohen’s κ		
	MISA	NOWAC	Combined	MISA	NOWAC	Combined
Mechanistic modeling	120/309 (39)	63/244 (26)	251/553 (48)	0.38	0.12	0.64
Linear regression models	142/308 (46)	91/232 (39)	341/540 (63)	0.59	0.46	0.81

**Figure 2:**
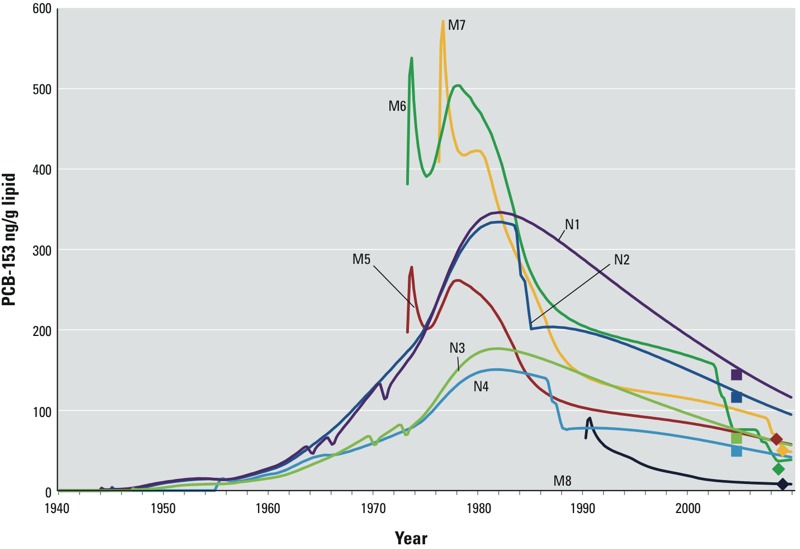
Predicted concentrations of PCB-153 for four MISA and four NOWAC women from their respective birth years until 2010 are displayed along with the concentrations measured at the time of sampling for each woman. MISA women (M, diamond-shaped markers) were sampled in 2007–2009, and NOWAC women (N, square-shaped markers) were sampled in 2005. N1: born in 1944, children born in 1963, 1965, and 1970; N2: born in 1945, child born in 1984; N3: born in 1947, children born in 1970 and 1973; N4: born in 1955, children born in 1987 and 1988; M5: born in 1973, no children; M6: born in 1973, children born in 2003 and 2007; M7: born in 1976, child born in 2008; M8: born in 1990, no children.

**Figure 3:**
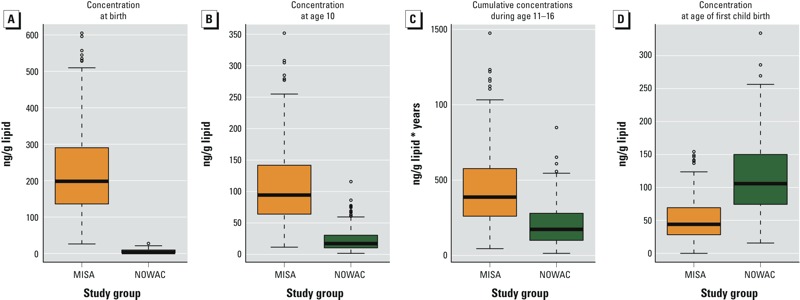
Person-specific predictions of concentrations of PCB-153 (*A*) at birth, (*B*) at 10 years of age, (*C*) for cumulative concentrations (product of concentration across time) during puberty (age 11–16 years), and (*D*) for concentrations at age of first childbirth are displayed separately for the two study groups. Boxes extend from the 25th to the 75th percentile, horizontal bars represent the median, whiskers extend 1.5 times the length of the interquartile range (IQR) above and below the 75th and 25th percentiles, respectively, and outliers are represented as points.

*Individual LBATs.* The CoZMoMAN- generated estimates of LBATs varied significantly between individuals. This variability is illustrated in Figure 2, in which measured concentrations at time of sampling are plotted along with CoZMoMAN-predicted concentrations of PCB-153 from birth until 2010 for selected MISA (*n* = 4) and NOWAC (*n* = 4) women. These women were chosen to represent different birth years and numbers of children. Predicted concentrations at different ages and cumulative exposures during puberty [represented by the area under the curve (AUC) for concentrations from 11 to 16 years of age] were derived for all individuals and are presented in Figure 3. Rank correlations and scatter plots including the predicted concentrations for the past and for the sampling time (as well as the measured values for rank correlations) are presented in the Supplemental Material, Figure S4 and Table S5. These results indicate that the magnitude of concentrations experienced by the study groups early in life varied between groups and that agreement between the measured and predicted values was best overall for the MISA group.

## Discussion

*Concentrations in pregnant and postmenopausal Norwegian women.* The median PCB-153 concentrations in the postmenopausal NOWAC women (Rylander et al. 2012) were roughly three times higher than those in the pregnant MISA women. The median age of the NOWAC women was 26 years older than that of the MISA women at the time of blood sampling (2005 and 2007–2009, respectively), which reflects the difference in median birth year (1949 and 1977, respectively). Higher concentrations of PCB-153 were expected in the older individuals because of the birth cohort effect, that is, the body burden in the older women is a remnant of much higher PCB exposure that they had experienced in the past (Nøst et al. 2013; Quinn and Wania 2012). The different dietary habits of the two study groups likely also contributed to the observed differences (Quinn et al. 2012). Indeed, consumption of marine food items was a predictor of PCBs in these women (Rylander et al. 2012; Veyhe et al. 2015), and the MISA women reported consuming less fish than the NOWAC women. Parity was generally higher in the NOWAC women than in the MISA women (17 and 212 nulliparous women, respectively); thus, concentrations of PCBs in NOWAC women could have been even higher if the loss of PCBs during pregnancy and breastfeeding could be taken into account (Norén and Meironyté 2000; Thomsen et al. 2010). The sample collection times in the MISA and NOWAC studies were 2007–2009 and 2005, respectively, and the influence of decreasing time trends of PCBs in humans during these years on the concentration differences between subject groups is likely modest.

*Predictive ability of CoZMoMAN for the study groups.* The median predicted PCB-153 concentrations in the MISA and NOWAC women were within 20% and 4%, respectively, of the measured values. The predicted concentrations were in good accord with those measured for PCBs 153 and 180, whereas the predicted values were slightly high for PCB-118 in both study groups and slightly low for PCB-138 in the NOWAC women. This last result may have been caused by the coelution of PCB-138 and PCB-163 during analysis of the NOWAC samples (these compounds were quantified separately in the MISA samples). Similar model discrepancies have been observed for PCB-118 in previous studies (Czub and McLachlan 2004; Nøst et al. 2013) and likely reflect inaccurate estimates of its metabolic degradation half-life (Czub and McLachlan 2004). Furthermore, the ranges of PCB-153 concentrations predicted by CoZMoMAN for the MISA and NOWAC women were similar to the measured concentration ranges, demonstrating that CoZMoMAN can reproduce realistic concentration ranges in women of different ages and states of parity.

*Person-specific predictions by CoZMoMAN.* The rank correlation of measured concentrations of PCB-153 and CoZMoMAN predictions was strong (*r_s_* = 0.67) when both groups were combined; however, the correlation was weak when both groups were ranked separately and was better for the MISA women (*r_s_* = 0.40) than for the NOWAC women (*r_s_* = 0.13). It should be noted that a perfect fit was not expected because the model assumes homogeneous background exposure for all individuals through food chain–related intake and does not incorporate spatial variation {does not discriminate between highly and marginally contaminated environments in Norway with regards to, e.g., PCB content in fish [Nilsen et al. 2011; Norwegian Scientific Committee for Food Safety (VKM) 2008]} and did not include individual input information for all model parameters (such as metabolism or body weight). The stronger correlations between measured and predicted concentrations for the MISA women than for the NOWAC women could be related to the age ranges in the two groups and to differences in internal exposure resulting from past behaviors associated with PCB exposure or elimination. In addition, the MISA women originated from northern Norway, whereas the NOWAC women were recruited from throughout all of Norway, and the CoZMoMAN predictions for the PCB content in fish may be more representative for northern Norway than for the country as a whole.

The prediction errors were largely explained by daily intake of fish, the year of the participant’s birth, and total duration of breastfeeding (see Supplemental Material, Table S3). Disregarding person-specific dietary intake in simulations increased the correlation between predictions and measurements for the MISA women, whereas this assumption led to an unrealistically small range of concentrations for the NOWAC women (see Supplemental Material, Figure S2). The stronger correlation observed for the MISA women could imply that although the simulations depended on the dietary intake of fish, this input variable also introduced some misreporting from individual questionnaires into the predictions. Nevertheless, the food-frequency questionnaires were considered to be of good quality, and the calculated dietary intake estimations were realistic (Rylander et al. 2012; Totland et al. 2012; Veyhe et al. 2012). Discrepancies between the predicted and measured concentrations were also associated with the breastfeeding variable (see Supplemental Material, Table S3); however, correlations between predicted and measured concentrations were better for women who had given birth and breast fed than for nulliparous women. Taken together, these findings imply that individual parameterization of each input variable is necessary to create contrasts in predicted concentrations, although it is likely that the input information also contributes to uncertainty in the model predictions.

Accounting for individual differences in parameters regarded as fixed by CoZMoMAN, such as body weight, could have improved the performance of the model. Lipid-normalized concentrations depend on the body’s lipid stores, and temporal changes in body lipid compartments have been suggested to influence the half-life of 1,1,1-trichloro-2,2-bis(4-chlorophenyl)ethane (DDT) in humans (Wolff et al. 2007). Thus, the observed association between reported body weights and discrepancies between predicted and measured concentrations could indicate that this factor should be individually parameterized in CoZMoMAN. Another contributing factor to individual variation in measured concentrations could be the varying metabolic capacities of PCBs (Shirai and Kissel 1996; Wolff et al. 1992), to which CoZMoMAN is sensitive (Quinn and Wania 2012), although individual parameterization is not feasible in this case.

*Predictors of concentrations in regression analyses.* Regression models were intended to identify predictors not accounted for by CoZMoMAN, and the best-fitted regression models for PCB concentrations included the following predictors: *a*) year of the participant’s birth and duration of breastfeeding for both study groups, and *b*) body weight and intake of fish liver and freshwater fish for the MISA women (see Supplemental Material, Table S4). Multivariate analyses in the MISA women indicated age, parity, body mass index, and dietary intake of freshwater fish, fatty fish, fish liver, and reindeer as significant predictors (Veyhe et al. 2015). MISA women who consumed seagull eggs were not considered in the evaluation of the performance of CoZMoMAN because this route of PCB exposure is not accounted for by the model; however, consumption of seagull eggs was a predictor of PCB concentrations in these women (Veyhe et al. 2015) as well as in a large cohort of pregnant Norwegian women (Caspersen et al. 2013). Intake of marine food was identified as a predictor of PCB concentrations in NOWAC women in a study employing a multivariate approach (Rylander et al. 2012); however, total fish intake was only borderline significant for the NOWAC women in the present study.

*Summarizing person-specific predictions.* Our evaluation of the predictions suggests that *a priori* estimates of person-specific PCB-153 concentrations from CoZMoMAN could represent valuable supplements to single measurements in exposure characterization. In the present study, it was possible to compare predictions and measurements for a large sample set that included questionnaire data of good quality. Both CoZMoMAN simulations and regression analyses provided good rank correlations and individual quartile categorization that agreed well with those of measured concentrations (weighted Cohen’s κ > 0.6) for both study groups. Rank correlations between measurements and predictions were strong only when the MISA and NOWAC women were regarded collectively, demonstrating that the model performed better when the targeted study group included a wide range of personal characteristics (e.g., both old and young people) as input information and thus also considered a wide spread of concentrations.

It is important to note that the main predictors for measured PCB concentrations indicated by statistical approaches were mechanistically accounted for by CoZMoMAN. Nevertheless, although fish intake was a predictor of PCB concentration, CoZMoMAN attributed too much of the inter-individual difference to variable fish intake. Furthermore, certain individual variation could not be explicitly addressed in CoZMoMAN, nor was it identified in statistical analyses of the information in questionnaires.

Person-specific prediction of PCB concentrations has been attempted previously, and the predictive ability of the regression approaches used in this study is similar to those reported for a Norwegian subpopulation with high intake of fish and game (Kvalem et al. 2012) and for elderly women in Sweden (Bergkvist et al. 2012). The ability of CoZMoMAN to reproduce individual PCB measurements is similar to those reported for a pharmacokinetic model based on dietary intake rates for Inuit adults (Sonne et al. 2014) and for a toxicokinetic model predicting three measurements throughout early childhood (Verner et al. 2013).

In this study, agreement between measured and predicted concentrations was better for predictions based on regression models than for predictions obtained from CoZMoMAN; however, this result was expected because regression models are constructed from the measurements themselves. Mechanistic modeling has the following advantages: *a*) It does not rely on measured concentrations as input, and *b*) it can avoid the pitfalls of statistical approaches in terms of causal relationships. From a modeling perspective, calculating all predicted concentrations within a factor of 10 of the measured concentrations and obtaining an acceptable overall ranking of individuals are remarkable achievements that lend further support for mechanistic modeling approaches. We believe that there is significant scientific and regulatory merit in mechanistically assessing the impact of changes in emissions on internal exposure at the individual level and across time. Nevertheless, our comparison between statistical and mechanistic approaches identified exposure pathways ignored by the latter approach that may be of significance to some individuals (e.g., consumption of seagull eggs). Because the model is parameterized for a specific region, further refinements may be required before it can be applied to other areas (e.g., Quinn et al. 2012).

*Single measurements and predicted LBATs.* The time-resolved feature of CoZMoMAN provides an expanded understanding of individual exposures with important perspectives on internal exposure on an individual basis when predicting LBATs. Figures 2 and 3 clearly show the large individual differences in LBATs and concentrations in study subjects at different ages; these figures also show that single measurements alone do not reveal inter-individual differences in past exposure. Evidently, conceptual understanding of variation and influential predictors as well as estimates of past concentrations are relevant for characterization of PCB exposure. Thus, such estimates may complement single measurements and may provide useful exposure measures for effect-related studies. Indeed, point estimates of PCB concentrations at birth, at 10 years of age, and at the age of first childbirth, as well as an estimate of cumulative exposure during puberty (11–16 years of age) were derived for both the MISA and the NOWAC women. The rank correlation was strong between the predicted individual concentrations at the time of sampling and the concentrations predicted for earlier in the lives of the MISA women (see Supplemental Material, Table S5). For the NOWAC women, corresponding correlations increased from birth until the age of first childbirth, that is, with decreasing time between sampling and the assumed period of exposure susceptibility. However, measured concentrations at the time of sampling appear to be only weakly correlated to the exposures predicted for possible time periods of high susceptibility earlier in life. Although temporal changes in concentration cannot be evaluated on the basis of the observations presented herein, key features of observed temporal trends of PCBs in humans across nearly 30 years have been reproduced by CoZMoMAN (Breivik et al. 2010; Nøst et al. 2013). Our results using CoZMoMAN nevertheless confirm the potential for estimations of past individual exposures to serve as exposure metrics in epidemiological studies, as suggested by Bachelet et al. (2010) and Verner et al. (2011).

With regards to the individual LBATs presented in this study, it is evident that the highest concentrations occurred in the older NOWAC women when they were adults, whereas the younger MISA women experienced peak exposures at birth. Breastfeeding of children by the NOWAC women had little impact on their LBATs when it occurred before the early 1980s (N1 and N3 in Figure 2), whereas breastfeeding in the 1980s caused concentrations in those older mothers to drop significantly (N2 and N4 in Figure 2). Breastfeeding also lowered concentrations in the MISA women (M6 and M7 in Figure 2) and changed the individual ranking of predicted PCB burdens in MISA women at the time of sampling (consider M6 and M7 relative to M5 in Figure 2). The influence of reproductive behavior on PCB-153 concentrations in mothers and in their children according to the CoZMoMAN model has been thoroughly discussed by Quinn et al. (2011).

## Conclusions

The CoZMoMAN model was able to reproduce group medians and ranges of PCB concentrations in pregnant and postmenopausal Norwegian women. Furthermore, ranking of individuals based on predictions and measurements was largely consistent, albeit weak when both study groups were regarded separately.

LBATs predicted by CoZMoMAN suggested large differences in the concentrations to which the MISA and NOWAC women had been exposed in the past. Mechanistic modeling can provide valuable information about PCB concentrations throughout an individual’s lifecycle that is a useful supplement to exposure characterization based on cross-sectional measurements of PCBs in blood.

Editor’s Note: Figure 1 showed untransformed concentrations in the Advance Publication. The revised figure shows log-transformed concentrations. It is included in this article.

## Supplemental Material

(452 KB) PDF
