# Supplementary material for: Estimating Time-Varying PCB Exposures Using Person-Specific Predictions to Supplement Measured Values: A Comparison of Observed and Predicted Values in Two Cohorts of Norwegian Women
Source: Environ Health Perspect. 2015 Jul 17;124(3):299–305. doi: 10.1289/ehp.1409191 (PMC4786984; doi:10.1289/ehp.1409191)
Supplement: (452 KB) PDF [file ehp.1409191.s001.acco.pdf]

Environ Health Perspect
DOI: 10.1289/ehp.1409191

Note to Readers: *EHP* strives to ensure that all journal content is accessible to all readers. However, some figures and Supplemental Material published in *EHP* articles may not conform to 508 standards due to the complexity of the information being presented. If you need assistance accessing journal content, please contact ehp508@niehs.nih.gov. Our staff will work with you to assess and meet your accessibility needs within 3 working days.

Supplemental Material

Estimating Time-Varying PCB Exposures Using Person-Specific Predictions to Supplement Measured Values: A Comparison of Observed and Predicted Values in Two Cohorts of Norwegian Women

Therese Haugdahl Nøst, Knut Breivik, Frank Wania, Charlotta Rylander, Jon Øyvind Odland, and Torkjel Manning Sandanger

Table of Contents

Table S1: Agreements of predictions for PCB-153 concentrations with measurements in separate simulations in which individual information was disregarded for one or several variables. Dietary intake input parameters were replaced by group medians and other parameters were replaced with zero for all individuals. In simulations including individual parameterisation of all variables the rank correlation was 0.40 and 0.13, and median discrepancy was +4.8 and -3.3 for MISA and NOWAC women, respectively,

Table S2: Summary of predicted concentrations of 3 PCBs from the mechanistic model CoZMoMAN and their comparisons those measured in MISA and NOWAC women. Pearson`s correlation was calculated based on log-transformed concentrations.

Figure S1: Measured serum concentrations (in ng/g lipid) of PCBs 118, 138 and 180 along with those predicted obtained from CoZMoMAN for the MISA (orange dots, *n*=310) and NOWAC (green squares, *n*=244) study subjects.

Table S3: Predictors of discrepancy between measured and predicted concentrations of PCB-153 for MISA and NOWAC women in linear regression models[a]. Models accounted for 50% and 56% of variations in model discrepancies for the MISA and NOWAC women, respectively.

Figure S2: Measured serum concentrations of PCB-153 along with those predicted (both in ng/g lipid) obtained from CoZMoMAN when assuming median daily intakes of fish, meat and dairy products for all individuals for the MISA (orange dots, n=310) and NOWAC (green squares, n=244) study subjects.

Table S4: Predictors of measured PCB-153 concentrations in linear regression models[a] of the MISA and NOWAC study subjects. The models for the MISA and NOWAC women accounted for 36% and 22% of variations in concentrations, respectively.

Figure S3: Measured serum concentrations of PCB-153 along with those predicted (both in ng/g lipid) from statistical linear regression models for MISA (orange dots, *n*=310) and NOWAC (green squares, *n*=233) study subjects.

Table S5: Rank correlation r_s between person-specific predictions of concentrations experienced in the past and at sampling time with the measured concentrations. The number of included women was 310 and 233 from the MISA and NOWAC studies, respectively.

Figure S4: Predicted serum concentrations of PCB-153 at sampling time along with those predicted (both in ng/g lipid) at birth, at age 10, cumulated during puberty (ng/g lipid*years) and at age of first child birth for MISA (orange dots, n=310) and NOWAC (green squares, n=244) women. Correlations are presented in Table S5.

Table S1: Agreements of predictions for PCB-153 concentrations with measurements in separate simulations in which individual information was disregarded for one or several variables. Dietary intake input parameters were replaced by group medians and other parameters were replaced with zero for all individuals. In simulations including individual parameterisation of all variables the rank correlation was 0.40 and 0.13, and median discrepancy was +4.8 and -3.3 for MISA and NOWAC women, respectively,

Static input value for:	Rank correlation r_s		Median discrepancy	
	MISA	NOWAC	MISA	NOWAC
Birth year	0.27	0.13	+5.31	-8.47
Duration of breastfeeding	0.23	0.08[a]	+11.0	-1.76
Age at childbirth	0.21	0.08[a]	+12.1	-0.8
No. of children	0.21	0.08[a]	+12.1	-0.8
Age at childbirth and duration of breastfeeding	0.21	0.08[a]	+12.1	-0.8
Dietary intake rates	0.46	0.25	+3.81	-6.13
Duration of breastfeeding and dietary intake rates	0.37	-0.21	+11.7	-1.0
Age at childbirth and dietary intake rates	0.29	-0.31	+11.8	-0.55
No. of children and dietary intake rates	0.29	-0.31	+11.8	-0.55
Age at childbirth, duration of breastfeeding and dietary intake rates	0.29	-0.31	+11.8	-0.55

Table S2: Summary of predicted concentrations of 3 PCBs from the mechanistic model CoZMoMAN and their comparisons those measured in MISA and NOWAC women. Pearson`s correlation was calculated based on log-transformed concentrations.

PCB cong.	Study group	Median prediction	Median discrepancy	Correlation r_s	Correlation r_p
118	MISA	8.31	3.76	0.31	0.33
	NOWAC	17.6	3.66	0.15	0.14
138	MISA	15.8	0.44	0.35	0.39
	NOWAC	35.7	-25.4	0.13ns	0.14
180	MISA	17.9	0.01	0.38	0.39
	NOWAC	58.3	-6.08	0.16	0.14

ns=not significant

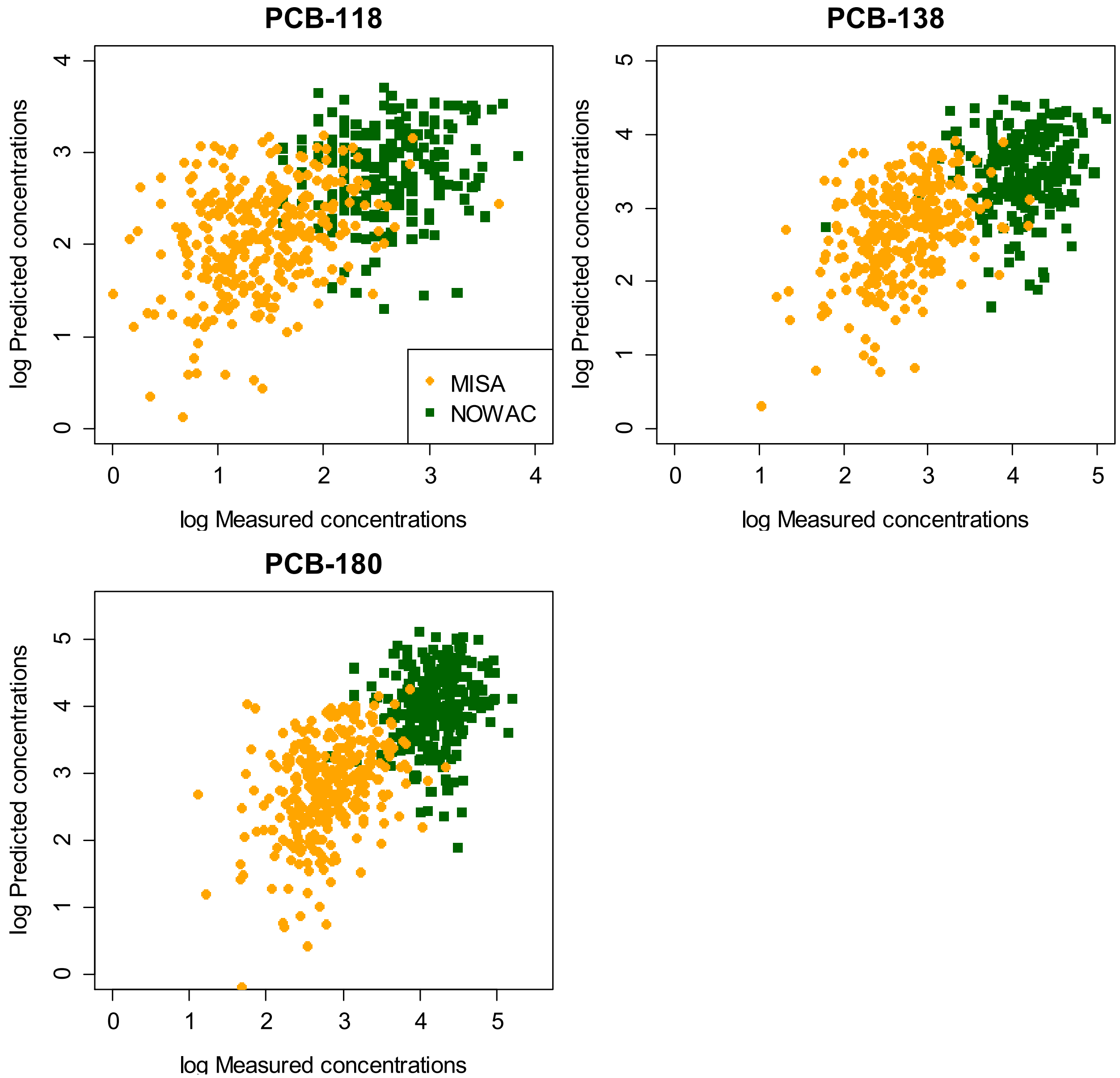

Figure S1: Measured serum concentrations (in ng/g lipid) of PCBs 118, 138 and 180 along with those predicted obtained from CoZMoMAN for the MISA (orange dots, *n*=310) and NOWAC (green squares, *n*=244) study subjects.

Table S3: Predictors of discrepancy between measured and predicted concentrations of PCB-153 for MISA and NOWAC women in linear regression models[a]. Models accounted for 50% and 56% of variations in model discrepancies for the MISA and NOWAC women, respectively.

Predictor	MISA			NOWAC		
	Coefficient estimate	SE[b]	p-value	Coefficient estimate	SE[b]	p-value
Birth year	0.76	0.18	1.75e-05	2.92	0.53	7.56e-08
Total breastfeeding	-0.88	0.18	1.68e-06	0.38	0.18	0.038
Intake of fish[c]	0.45	0.03	<2e-16	0.83	0.05	<2e-16
Body weight	0.22	0.06	5e-04			

[a]Intercepts for MISA and NOWAC models were significant. Further, levels of significance of predictors were as follows: '***'=p<0.001; '*'=p<0.05.
[b]SE = Standard error of estimate.
[c]Intakes represents summed fish intakes in g fresh weight/day.

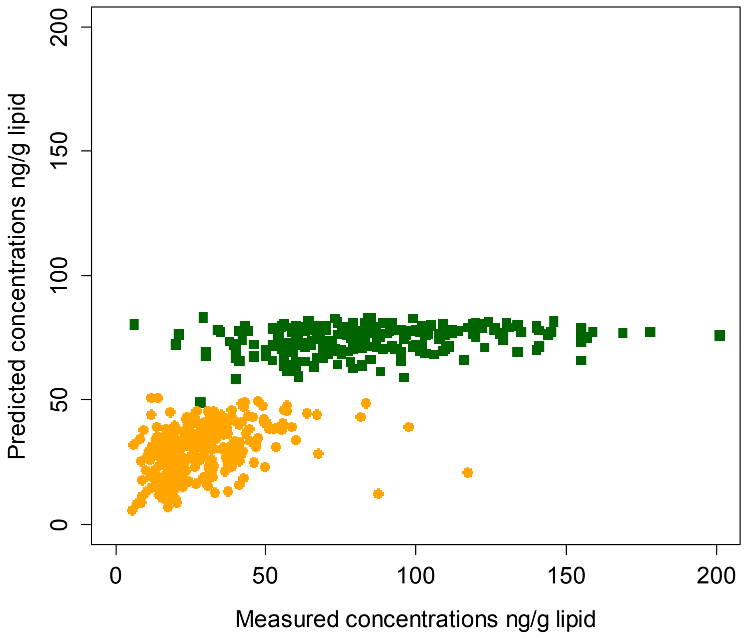

Figure S2: Measured serum concentrations of PCB-153 along with those predicted (both in ng/g lipid) obtained from CoZMoMAN when assuming median daily intakes of fish, meat and dairy products for all individuals for the MISA (orange dots, n=310) and NOWAC (green squares, n=244) study subjects.

Table S4: Predictors of measured PCB-153 concentrations in linear regression models[a] of the MISA and NOWAC study subjects. The models for the MISA and NOWAC women accounted for 36% and 22% of variations in concentrations, respectively.

	MISA			NOWAC		
Predictor	Coefficient estimate	SE[b]	p-value	Coefficient estimate	SE[b]	p-value
Birth year	-1.49	0.15	<2e-16	-2.86	0.51	8e-08
Duration of breastfeeding (mths)	-1.29	0.15	5e-16	-0.95	0.18	3e-07
Body weight (kg)	-0.21	0.05	0.0001			
Intake of fish liver	11.0	3.24	0.0008			
Intake of freshwater fish	1.23	0.27	6e-06			
Intake of summed fish				0.09	0.05	0.06

[a]Intercepts for MISA and NOWAC models were significant. Further, levels of significance of predictors were as follows: '***'=p<0.001; '**'=p<0.01; '*'=p<0.05; '.'=p<0.1.
[b]SE = Standard error of estimate

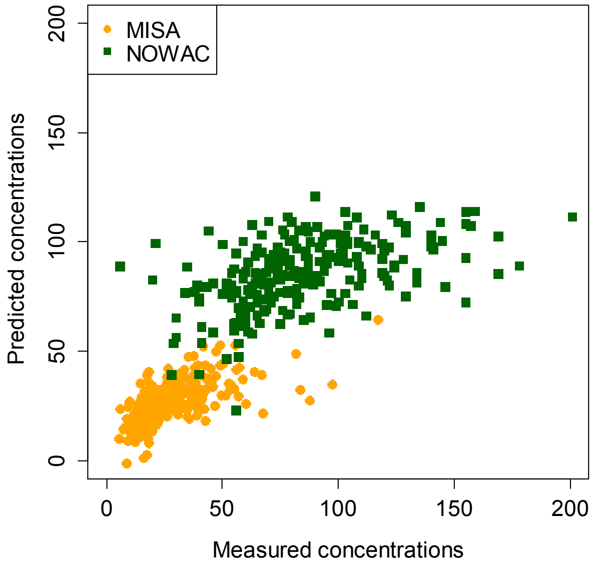

Figure S3: Measured serum concentrations of PCB-153 along with those predicted (both in ng/g lipid) from statistical linear regression models for MISA (orange dots, *n*=310) and NOWAC (green squares, *n*=233) study subjects.

Table S5: Rank correlation r_s between person-specific predictions of concentrations experienced in the past and at sampling time with the measured concentrations. The number of included women was 310 and 233 from the MISA and NOWAC studies, respectively.

**Predictions of concentrations**	**Prediction at sampling time**		**Measurement**	
	**MISA**	**NOWAC**	**MISA**	**NOWAC**
At birth	0.68	0.27	0.05[a]	-0.32
At 10 years	0.65	0.39	0.29	-0.31
During puberty	0.66	0.44	0.29	-0.29
At age of first child birth	0.60	0.72	0.38	-0.13

[a]Correlation was not significant

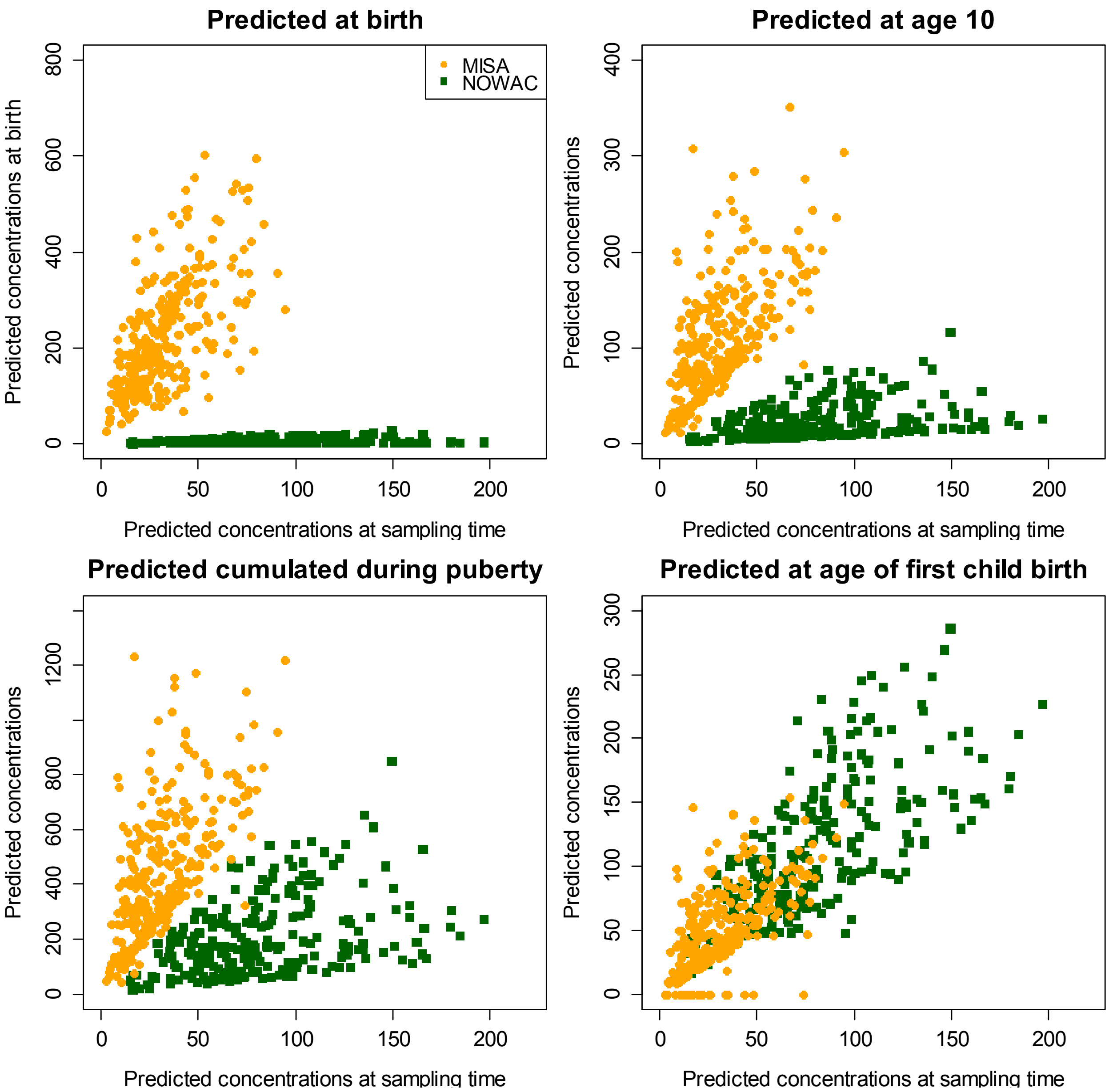

Figure S4: Predicted serum concentrations of PCB-153 at sampling time along with those predicted (both in ng/g lipid) at birth, at age 10, cumulated during puberty (ng/g lipid*years) and at age of first child birth for MISA (orange dots, n=310) and NOWAC (green squares, n=244) women. Correlations are presented in Table S5.
